# Searching for the optimal tDCS target for motor rehabilitation

**DOI:** 10.1186/s12984-019-0561-5

**Published:** 2019-07-17

**Authors:** Isadora Santos Ferreira, Beatriz Teixeira Costa, Clara Lima Ramos, Pedro Lucena, Aurore Thibaut, Felipe Fregni

**Affiliations:** 1 Neuromodulation Center, Spaulding Rehabilitation Hospital, Harvard Medical School, Boston, USA; 2 Coma Science Group, GIGA-Consciousness, University of Liege, Liege, Belgium; 3 Neuromodulation Center, Spaulding Rehabilitation Hospital and Massachusetts General Hospital, Department of Physical Medicine and Rehabilitation, Harvard Medical School, 79/96 13th Street, Charlestown, MA 02129 USA

**Keywords:** Transcranial direct current stimulation, Pain modulation, Motor rehabilitation, Non-invasive brain stimulation

## Abstract

**Background:**

Transcranial direct current stimulation (tDCS) has been investigated over the years due to its short and also long-term effects on cortical excitability and neuroplasticity. Although its mechanisms to improve motor function are not fully understood, this technique has been suggested as an alternative therapeutic method for motor rehabilitation, especially those with motor function deficits. When applied to the primary motor cortex, tDCS has shown to improve motor function in healthy individuals, as well as in patients with neurological disorders. Based on its potential effects on motor recovery, identifying optimal targets for tDCS stimulation is essential to improve knowledge regarding neuromodulation as well as to advance the use of tDCS in clinical motor rehabilitation.

**Methods and results:**

Therefore, this review discusses the existing evidence on the application of four different tDCS montages to promote and enhance motor rehabilitation: (1) anodal ipsilesional and cathodal contralesional primary motor cortex tDCS, (2) combination of central tDCS and peripheral electrical stimulation, (3) prefrontal tDCS montage and (4) cerebellar tDCS stimulation. Although there is a significant amount of data testing primary motor cortex tDCS for motor recovery, other targets and strategies have not been sufficiently tested. This review then presents the potential mechanisms and available evidence of these other tDCS strategies to promote motor recovery.

**Conclusions:**

In spite of the large amount of data showing that tDCS is a promising adjuvant tool for motor rehabilitation, the diversity of parameters, associated with different characteristics of the clinical populations, has generated studies with heterogeneous methodologies and controversial results. The ideal montage for motor rehabilitation should be based on a patient-tailored approach that takes into account aspects related to the safety of the technique and the quality of the available evidence.

## Introduction

Transcranial Direct Current Stimulation (tDCS) is a non-invasive brain stimulation technique which delivers a constant electric current over the scalp to modulate cortical excitability [1–3]. Different montages of tDCS may induce diverse effects on brain networks, which are directly dependent on the electrodes positioning and polarity. While anodal tDCS is believed to enhance cortical excitability, cathodal tDCS diminishes the excitation of stimulated areas, and these electrodes montages define the polarity-specific effects of the stimulation [4–6]. Due to the effects of tDCS on modulating cortical excitability, especially when applied to the primary motor cortex [2], this method of brain stimulation has been intensively investigated for motor function improvement both in healthy subjects [7, 8] and in various neurological pathologies [9, 10]. Neurological conditions that may obtain benefits from the use of tDCS include Stroke [11–14], Parkinson’s disease [15], Multiple Sclerosis [16, 17], among others.

The mechanisms of action underlying the modulation of neuronal activity induced by tDCS are still not completely understood. However, studies have demonstrated that the electric current generated by tDCS interferes in the resting membrane potential of neuronal cells, which modulates spontaneous brain circuits activity [1–3]. Some studies have suggested that tDCS could have an effect on neuronal synapsis’ strength, altering the activity of NMDA and GABA receptors, thus triggering plasticity process, such as long-term potentiation (LTP) and long-term depression (LTD) [18, 19]. The long-term effects of tDCS are also thought to be associated to changes in protein synthesis and gene expression [20, 21]. Additionally, neuroimaging study showed blood flow changes following stimulation, which may be related to a direct effect of tDCS over blood flow, with an increase in oxygen supply on cortical areas and subsequent enhancement of neuronal excitability [22]. Given these mechanisms, tDCS seems to be a potential valuable tool to stimulate brain activity and plasticity following a brain damage.

The advantages of using tDCS include its low cost, ease of application, and safety. To date, there is no evidence of severe adverse events following tDCS in healthy individuals, as well as in patients with neurological conditions, such as stroke [23, 24]. Among the potential side effects presented after this type of stimulation, the most common ones consist of burn sensation, itching, transient skin irritation, tingling under the electrode, headache, and low intensity discomfort [25]. As serious and irreversible side effects have not been reported, tDCS is considered a relatively safe and tolerable strategy of non-invasive brain stimulation.

The modifications of physiological and clinical responses induced by tDCS are extremely variable, as this type of stimulation can induce both adaptive or maladaptive plastic changes, and a wide spectrum of tDCS parameters influence the effects of this technique. Electrodes combination, montage and shape can easily interfere in the enhancement or inhibition of cortical excitability [6, 26]. Other parameters that may influence these outcomes include current intensity, current flow direction, skin preparation, and stimulation intervals [3, 27, 28] . In addition, in clinical populations, the heterogeneity of the brain lesions can also influence the inconsistency in tDCS effects [29]. Despite the goal of tDCS of modulating cortical areas by using different parameters, some studies have showed that, by altering cortical excitability, the electrical field could reach subcortical structures, such as basal ganglia, due to brain connections between cortical and subcortical areas [30–33]. This potential effect on deeper brain structure has supported the broad investigation of tDCS in various disorders, even if the cortical region under stimulating electrode is not directly linked to the neurological condition being investigated. Indeed, the current variable and moderate effect sizes from clinical tDCS studies in stroke encourage researchers to test alternative targets to promote motor recovery in this condition.

In this review, we discuss evidence on the application of four different tDCS montages to promote and enhance motor rehabilitation: [1] anodal tDCS ipsilateral and cathodal tDCS bilateral, [2] combination of central and peripheral stimulation, [3] prefrontal montage and [4] cerebellar stimulation.

## Basic model: anodal ipsilesional M1 and cathodal contralesional M1

The continuous search for the optimal placement of tDCS electrodes has been one of the main topics discussed in research studies over the years [11, 34–36]. In fact, one of the reasons for the lack of effectiveness in early tDCS studies was inadequate electrode montages which influenced the amount of significant current being injected in cortical areas [34]. As additional elements may also influence the efficacy of the stimulation, such as the intensity of the current, duration and target of the stimulation [37], as well as elements involved with physiopathological aspects of a certain condition, such as severity [35], defining the most effective stimulation parameters and how to promote changes that outlast the stimulation period becomes fundamental. The application of tDCS is based on the premise that a low-intensity direct current, delivered through two electrodes, can facilitate either the depolarization (anodal) or the hyperpolarization (cathodal) of underlying brain regions based on Electroencephalography (EEG) mapped sites (e.g. Primary motor cortex – M1) [38], thus guiding brain plasticity for the recovery of symptoms and after-effects of neurological conditions.

Considering the variety of tDCS’ existing configurations and its applicability in different fields (e.g. attention, cognition, motor recovery), it is possible to correlate electrodes montage with the brain region that would generate benefits and the most effective changes when activated or inhibited [39]. For instance, when it comes to motor recovery, that corresponding area is the motor cortex [34]. Studies have shown that, in healthy subjects, anodal tDCS over M1 facilitates neuronal firing and promotes cortical excitability, which also seems to be correlated with an increase in the motor evoked potential (MEP) amplitude [39]. Cathodal tDCS, on the other hand, inhibits neuronal excitability. In addition, a few studies have also showed that, in healthy subjects, bilateral stimulation promotes significant improvements in the non-dominant hand and is also associated with a larger effect on motor function as compared to unilateral stimulation [34]. By taking these findings into a clinical scenario, it is possible to assume that patients who suffer from hemiparesis or have motor deficits after a stroke, for instance, could benefit from noninvasive brain stimulation using tDCS electrodes in one of these configurations. In fact, tDCS application in motor domain for stroke patients has shown to be effective in enhancing performance in functional tasks and muscle force [36].

The mechanisms and neural pathways underlying the recovery process in stroke patients is still uncertain. However, an early recovery has been associated with neuroplasticity, due to regenerative phenomena such as axonal and dendritic sprouting, and brain reorganization, as observed in functional magnetic studies [40, 41]. These studies have shown that, regarding stroke, there is an increased bihemispheric activation when the affected body part is moved, thus aligning with the idea of a brain reorganization existence, which could represent either a recovery or a maladaptive process [42]. The reactivation or overactivation of certain brain areas due to maladaptation after a stroke corresponds to an imbalance of interhemispheric inhibition. This imbalance is a result of the inhibition from the unaffected hemisphere (ipsilateral to the affected hand/arm) onto the lesional hemisphere, which interferes with the recovery process, increasing its duration, prolonging the need of a therapy and, consequently, harming life quality.

**Fig. 1:**
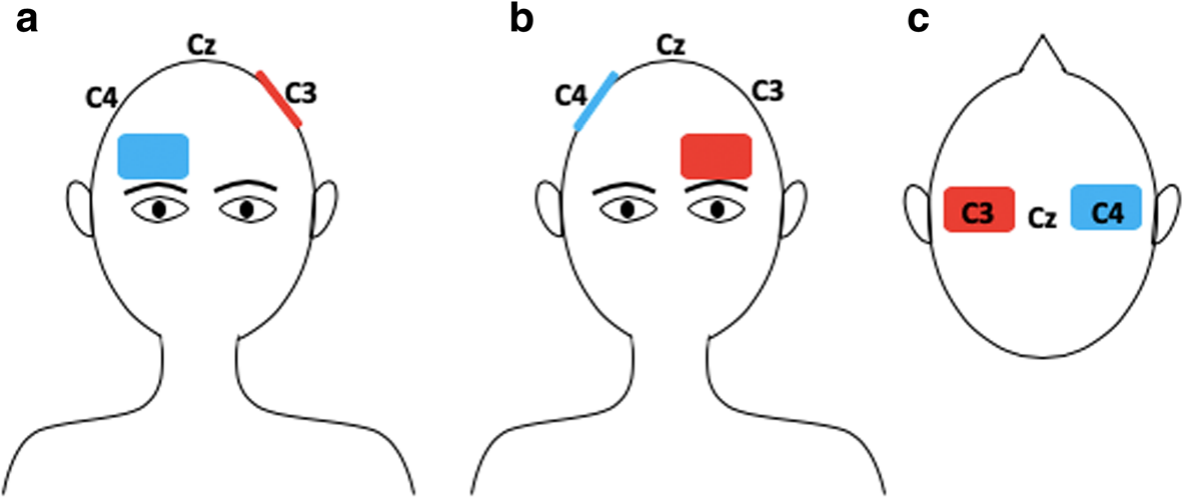
Motor cortex stimulation in a scenario where the left hemisphere was lesioned. Figure **a** Anodal stimulation of left primary motor cortex: anode over the left M1 and cathode over the right supraorbital region. Figure **b** Cathodal stimulation of right primary motor cortex: cathode over the right M1 and anode over the left supraorbital region. Figure **c** Bilateral stimulation: anode over the affected hemisphere (left) and cathode over the non-affected hemisphere (right)

This imbalanced inhibition is the hypothesis that supports the use of tDCS as an alternative therapeutic approach for post-stroke rehabilitation, thus applying anodal tDCS to the lesional hemisphere as to increase its excitability, cathodal tDCS to the unaffected hemisphere as to inhibit its inhibition over the affected hemisphere [42], and bilateral stimulation as to achieve the effects of both types of unilateral stimulation at the same time. Although further research is still needed, several studies involving stroke patients have shown that anodal tDCS over M1 of the lesioned hemisphere can improve motor cortex and hand motor tasks [43], especially if conducted for 7 days, which would prolong the effects [12]. This montage consists in placing the anode over the M1 ipsilateral to the affected side and the cathode over the supra orbital region contralateral to the affected side, as shown in Fig. 1a. As a result, there is an increase of cortical excitability in the affected hemisphere. This rationale is the first main therapeutic strategy proposed by the interhemispheric competition model, which states that the unaffected motor region exerts an inhibitory activity over the affected motor cortex, thus limiting post stroke motor recovery [44]. Yet, several studies have also shown performing cathodal tDCS over M1 (Fig. 1b), which consists in placing the anode in the ipsilesional M1 and the cathode in the contralesional M1, can improve motor learning [11, 45, 46]. These studies corroborate with the second therapeutic strategy proposed by the interhemispheric competition model. It suggests that decreasing activity in the healthy hemisphere (downregulation) using cathodal tDCS may lead to a decreased inhibition over the affected hemisphere due to transcallosal inhibition [39]. Hence, facilitating motor recovery.

Finally, bilateral stimulation consists in placing the anode over the lesioned hemisphere and the cathode over the healthy hemisphere, as shown if Fig. 1c. By comparing this electrode montage with unilateral stimulation, Mahmoudi et al. have observed in their study that motor function improvements generated from bilateral stimulation cannot be larger than by what is induced from unilateral stimulation as subjects had similar effects when at least one electrode placement in common was used [34]. A potential explanation is that the second M1 electrode might not have an additive effect. Vines at al, on the other hand, have showed that bilateral stimulation is associated with larger effects on finger-sequencing task with the non-dominant hand as compared with unilateral stimulation [47]. In spite of having better results related with bilateral stimulation, it is worth mentioning that this study was conducted in healthy subjects while the one from Mahmoudi et al. was conducted in stroke patients. Therefore, results regarding the additive effects of bilateral stimulation and its potential benefits over unilateral stimulation are still controversial.

Although a few studies have already tested the effects of different electrodes montages for tDCS application in stroke patients, recent studies have shown that other aspects may interfere in patients’ response to M1 stimulation. Some of these aspects include stroke duration (whether it is chronic or acute), patient’s age, stroke location and especially baseline motor function [48]. A systematic review from Bertolucci et al. concluded that transcallosal inhibition (TCI) as a mechanism for stimulation of the contralesional hemisphere seems to depend on baseline motor function, as data gathered mostly from chronic patients showed that suppressing the activity of the contralesional hemisphere could be beneficial for patients with good residual motor function and strong TCI, but not for those with poor motor function and weak TCI [48]. The articles included in their review showed controversial results when it comes to the previous idea that higher activity in the contralesional hemisphere after a stroke leads to greater amounts of TCI exerted on the ipsilesional hemisphere. While a few were in favor of this idea, others argued that there are still better models to be proposed. Therefore, given the variation between study results, it is possible to assume that further research is fundamental, and that baseline function and measure of TCI should be considered for patient stratification in future clinical trials.

Another aspect worth considering is how effective the stimulation is, given the amount of current reaching the targeted brain area. The existence of different layers (with unique conductance) though which the current has to pass before reaching the targeted area (e.g. skull, meninges, cerebral spinal fluid (CSF), and ultimately the cortex) might result in significant dispersion as a consequence. In stroke patients, for instance, Wagner et al. highlight that a number of physiologic changes occur in the brain tissue, thus altering electric response to stimuli [49]. These changes can be explained by an inflammatory response in the infarction region which is responsible for the replacement of necrotic brain tissue for CSF. The level of replacement depends on the degree of damage. As a result, the CSF influx represents a six-fold increase in conductance in the infarction region and a significant modification of both geometry and conductive matrix of the region [49]. Therefore, it is crucial to take into account the physiological changes related with this condition when it comes to deciding stimulation parameters and techniques.

Finally, after considering all the pros and cons of this type of stimulation for stroke patients as well as the aspects that might interfere with study results, it is possible to raise a question: Have researchers truly found optimal targets and stimulation strategies for motor recovery so far? Although there are multiple alternatives of stimulation targets for improving motor function, intrinsic mechanisms and neurophysiological effects of the techniques still need to be explored.

## Therapy combination: central and peripheral stimulation

The treatment of chronic conditions as well as the management of its after effects have not always been easy tasks. In fact, there are several already approved treatments to improve motor recovery, especially in patients that have suffered a stroke [50–52]. Yet, as these available treatments (pharmacotherapy and physical therapy, for instance) are not always effective for all individuals, somatosensory stimulation combined with brain stimulation has become a possible therapeutic alternative, especially for motor recovery and pain relief. According to different studies, the possibility to combine the effects of central stimulation as well as peripheral stimulation, can provide significant benefits to the patient, thus improving quality of life [52]. However, as the mechanistic aspects of each intervention applied separately and concomitantly are not completely understood, further investigation is still essential.

### Peripheral stimulation

The benefits of performing peripheral stimulation have already been widely investigated for different conditions, such as rheumatoid arthritis (RA) [53], stroke [50–52], chronic pain [54, 55] and others. One of the most traditional techniques of peripheral stimulation is Transcutaneous Electrical Stimulation (TENS), a safe and non-invasive device which aims to stimulate the nerves for therapeutic purposes. Although its therapeutic effects have been proven, how this device affects neural pathways is still not completely clear. It is suggested that this type of electroanalgesia is produced according to the gate control theory [56], which states that physical pain is not a direct result of the activation of pain receptor neurons. Instead, its perception is modulated by interaction between different neurons. Thus, through different frequencies and intensities, TENS is able to modify the interaction between neurons and consequently alter pain perception.

In stroke patients, peripheral stimulation alone has shown to be a promising technique to enhance swallowing [57], pinch force [58], use-dependent plasticity [59], and ADL-like tasks [60, 61]. The physiological pathway of peripheral stimulation to modulate motor training in subcortical stroke patients is still not entirely comprehended, as most of the available studies have exclusively measured performance right after the stimulation. Nonetheless, there is evidence that the reorganizational process induced by peripheral stimulation is different across patients, mostly depending on the lesion brain location (cortical or subcortical area) [62], which may explain different results showed in clinical trials.

Celnik et al. showed improvement in ADL-like activities in subcortical stroke patients when peripheral stimulation was applied before motor training [61]. Hence, it is believed that when the stimulation is applied to peripheral nerves of body members with low motor function, there may exist an increase in corticomotor excitability [59, 63]. In addition Hope Pan et al. showed that peripheral stimulation in stroke subjects leads to motor function improvement and increased corticomuscular coherence, a measurement of synchronization level between EEG and electromyography [64]. There is still a great deal to learn as to understand how peripheral stimulation leads to motor function improvement; however, it is clear that it does modulate motor related neural networks neuroplasticity.

Other studies, however, suggest that peripheral stimulation may not be effective when it comes to modulating neuroplasticity, and its effects are not strong enough to reach encephalic levels [54]. Thus, in order to obtain new and strong brain connections as to facilitate leaning and ultimately induce a long-lasting motor recovery, the use of TENS as a single therapeutic approach may not be the most adequate option. As mentioned above, over time, studies have been trying to find the best combination and application between therapies to optimize motor recovery.

### Central stimulation

**Fig. 2:**
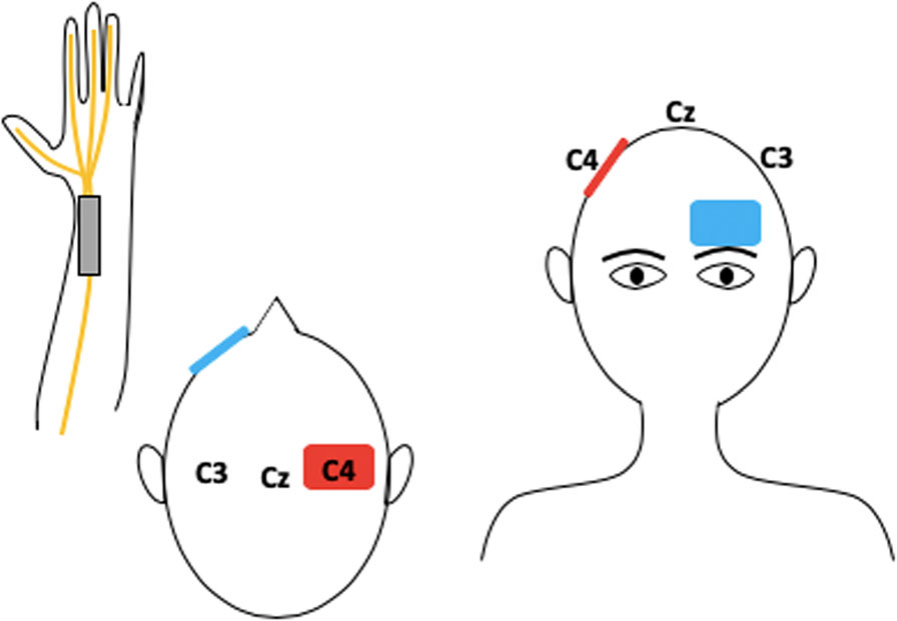
Left median nerve stimulation combined with tDCS. Anodal stimulation of the right motor cortex (C4) with the reference electrode over the contralateral supraorbital region

When applied over a certain motor cortical area, through different montages, tDCS is able to modulate a response and enable cortical reorganization. Thus, it is suggested that, by combining this type of stimulation with peripheral stimuli, it would be possible to enhance the effects of each intervention individually and, as a consequence, achieve faster and long-lasting results [52]. An example of the application of both peripheral and central stimulation is represented in Fig. 2. Additionally, as it has been proposed that tDCS is able to alter sodium and calcium channels as well as NDMA-receptor’s activity while peripheral stimulation exerts more influence over GABAergic interneurons and less modulations of NDMA-receptor. Therefore, it is possible to suggest that central and peripheral stimulation have synergistic effects in neuromodulations tasks and cortical excitability [52].

Several studies have shown important results regarding the combination of central and peripheral stimulation. For instance, Boggio et al. have proven that there was pain relief in patients with chronic pain, especially low back pain, with the use of tDCS and TENS [54, 55]. Although, the authors described that the ideal number of sessions is still unknown, bihemispheric tDCS in combination with peripheral sensorimotor activity led to substantial functional improvements, especially after the first 5 days of intervention [50]. Finally, Chalah et al. have also investigated the stimulation combination in patients that had essential tremor, obtaining positive results.

Therefore, although there has been a significant advance in research involving this topic, its status is still preliminary especially in the motor recovery field. This can be explained by the fact that most studies are preliminary and have a limited sample size, thus not accounting for different individual characteristics that may alter the final results. Also, most studies investigate patients that have suffered a stroke, consequently only dealing with the physiopathological aspects of this condition. Hence, it is fundamental that researchers explore other conditions that may also lead to motor deficits, identifying the mechanisms involved and observing how these patients respond to the combination between stimulations.

## Prefrontal montage for attention, cognition and motor recovery

The effects of tDCS are directly dependent on the brain area under stimulation and searching for an optimal target to promote motor or cognitive rehabilitation has become the spotlight of recent studies. Among the current targets under investigation, the prefrontal cortex holds promise for this purpose due to its connectivity with multiple brain regions, such as the primary motor cortex and structures of the attentional system [65].

**Fig. 3:**
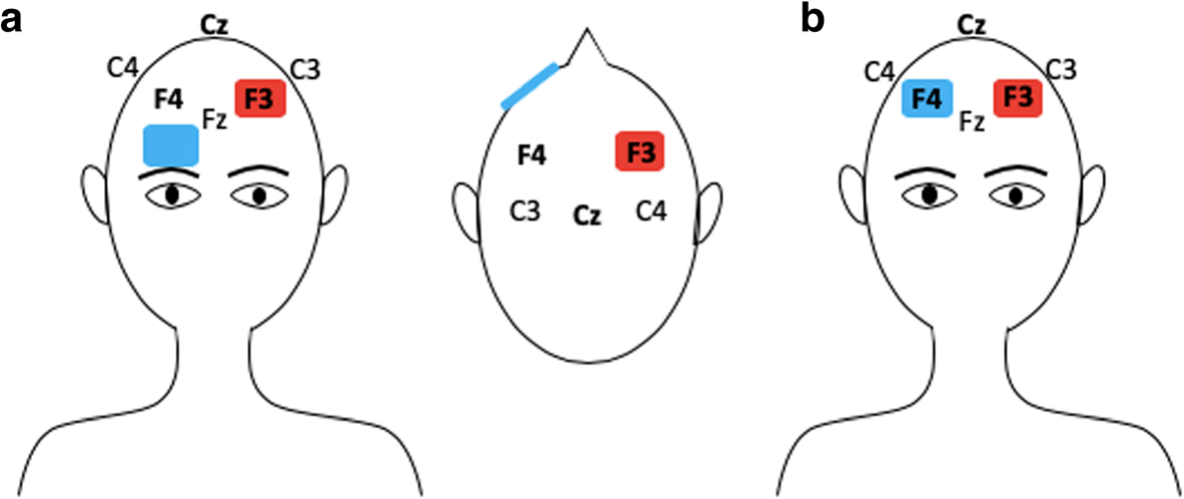
Anodal stimulation of left dorsolateral prefrontal cortex (DLPFC). Figure **a**: reference electrode positioned over the contralateral supraorbital region. Figure **b**: reference electrode positioned over the right DLPFC

The prefrontal cortex is known to actively participate in the control of cognitive performance, including attention and executive functions [66]. Studies on neurological and psychiatric conditions, such as Parkinson’s Disease and Depression, have focused on investigating the effects of anodal tDCS over the dorsolateral prefrontal cortex (DLPFC) due to its high connectivity with brain domains involved with mood changes, emotion regulation and cognition [67]. Furthermore, a recent study of Pope et al. has suggested that anodal stimulation over the left DLPFC (Fig. 3a) have a significant impact on verbal working memory performance during high demanding tasks by facilitating cognition [68]. Such argument corroborates with another recent clinical trial which have showed that anodal tDCS to the left DLPFC enhances executive functions, causing no harms to motor symptoms [69]. Although the majority of tDCS studies focuses on stimulation effects over the left DLPFC, there is evidence that both right and left prefrontal cortex have a role on cognitive functioning. Additionally, Gbadeyan et al. provided evidence for enhancement of adaptive cognitive control after stimulation of the left and right DLPFC, without predominance of either hemispheres [70].

Further research is required for exploring the effects of brain stimulation over both motor and prefrontal cortex, as cognitive functions (e.g. attention and memory) influence motor rehabilitation through complex neurophysiological mechanisms which are not fully comprehended until now. In fact, as highlighted in a review by Rossi et al., the prefrontal cortex influences individuals’ ability to switch attentional control according to task demands [71], which emphasizes its potential role in the process of motor rehabilitation. Thanks to the development of more sophisticated stimulation tools, it is now possible to target different brain regions using multichannel montages. The stimulation of the motor and the prefrontal cortices simultaneously has been recently considered as a promising technique for enhancing executive function (planning and execution). As an example, a recent study by Dagan et al. evaluating the effects of (a) simultaneous primary motor cortex and left dorsolateral prefrontal cortex stimulation, (b) primary motor cortex stimulation only, and (c) sham stimulation, has demonstrated that combining motor and prefrontal stimulation could reduce freezing of gait and improve mobility in patients with Parkinson disease [72]. Although this approach still needs to be explored in post-stroke individuals, the possible beneficial effects on neurodegenerative and attentional disorders, especially through the stimulation of both cognitive and motor function brain areas, may lead to important functional improvement for stroke rehabilitation [10].

Lateral and medial prefrontal cortex, as well as the anterior cingulate cortex and superior parietal lobule, seem to be correlated with attentional state [71]. As the prefrontal cortex often participates in the top-down control of attention, tDCS’ effects over this brain area may be associated with the activation and improvement of attention tasks. Clinical trials have reported that executive attention may be enhanced by 20 min anodal tDCS over left DLPFC not only in healthy individuals, but also in patients with fibromyalgia [17, 73]. These findings complement the results of other studies in the field which demonstrated that left DLPFC activation may contribute to modifying attentional bias [74]. For anxious individuals that suffers with biased attention, these results support an alternative therapy with tDCS for modulating attention to threat. Furthermore, Miler et al. has provided evidence that anodal tDCS over left DLPFC facilitated attentional disengagement, directly influencing emotional attention, while right DLPFC stimulation diminished it [75]. As the effects of tDCS to the prefrontal cortex on attention is a relatively new scope of investigation, the exact outcome from the right and left DLPFC stimulation is still to be fully comprehended.

The application of tDCS over the primary motor cortex (M1) is a technique commonly used for augmenting motor recovery; however, there is evidence that the stimulation of DLPFC in conjunction with M1 (Fig. 3b) may expand this positive effect on motor function [76]. This affirmation aligns with the results of a recent clinical trial which demonstrated that the activation of both M1 and DLPFC by anodal tDCS may increase M1 excitability [77]. With that being said, it may be presumed that DLPFC is functionally connected with M1 through different circuits, justifying the potential of DLPFC stimulation to contribute with motor rehabilitation. In addition, it is suggested that anodal tDCS of M1 and DLPFC concomitantly may provide a greater increase in corticospinal excitability than M1 stimulation alone, which is another evidence of DLPFC stimulation potential to expand tDCS effects on motor recovery [78]. This tDCS application was further tested in a trial involving motor impaired individuals after acute ischemic stroke, indicating that M1-DLPFC stimulation also resulted in superior motor function [76]. Despite the significant evidence on the influence of prefrontal cortex activation for motor rehabilitation, the underlying mechanisms of this technique still need to be explored, especially in larger and long-term clinical trials.

## Cerebellar stimulation

**Fig. 4:**
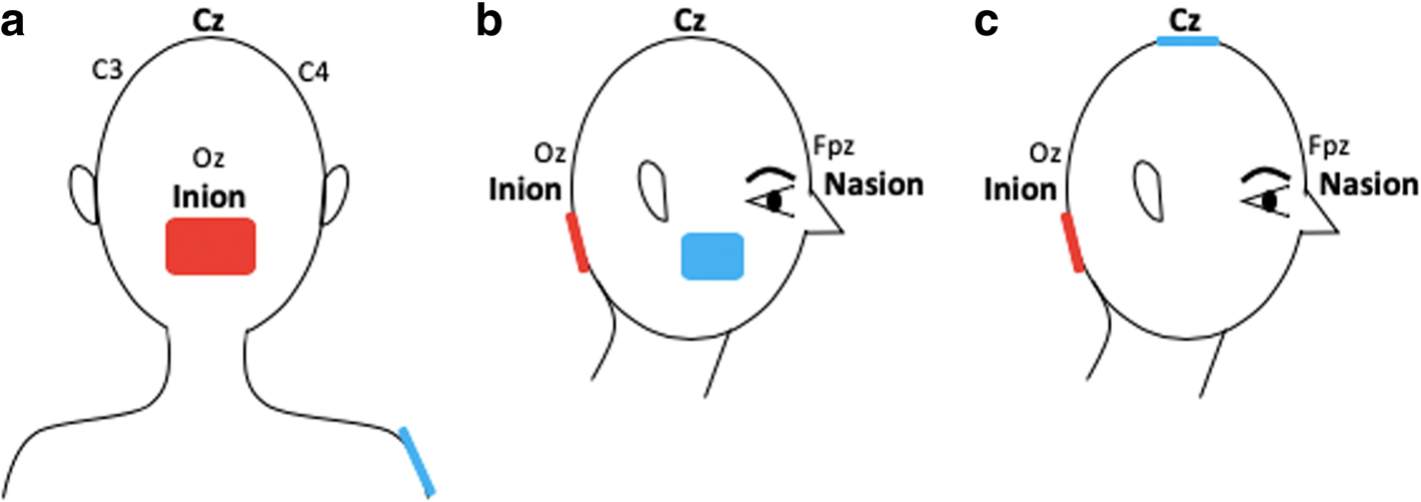
Bilateral cerebellar hemispheres stimulation. The active electrode is placed 1 to 2 cm below the inion. Figure **a**: anodal stimulation of the cerebellum with the reference electrode placed over the right shoulder. Figure **b**: anodal stimulation of the cerebellum with the reference electrode placed over buccinator muscle. Figure **c**. anodal stimulation of cerebellum and reference electrode over Cz

Beside cortical areas linked to motor function, the cerebellum is another sub-cortical region critical for various aspects of motricity, such as gait, balance and fine motor functions. In addition to its role in motor functions, studies have shown its implication in cognition, including motor learning [79, 80]. Given the role of the cerebellum in various aspects of motion, it has been recently seen as a potential target to stimulate in order to improve motor recovery following a stroke [81]. In this condition, stimulating the cerebellum can be used to improve its functions in case of a lesion within this region, or, on the other hand, be used to improve the recovery of impairments due to a supratentorial stroke via the stimulation of a non-damaged area. This second approach, is even more interesting knowing the recent findings which show that, in case of severe brain lesions, it may be pointless to target the damaged brain area as a partial metabolic and grey matter preservation is required for patients to clinically respond to tDCS [82]. Besides, motor deficits in stroke can also be due to crossed cerebellar diaschisis [83] as a consequence of supratentorial ischemic stroke. As for other forms of cortical diaschisis, it can become chronic with a prolonged reduction of blood flow and decrease of spontaneous Purkinje cells’ activity [84]. In this context, NIBS represent an attractive option given its effect on both cerebral blood flow and neural spiking activity [85]. For all these reasons, tDCS targeting this brain region seems very promising to enhance motor recovery following a stroke. A few examples of electrode montages are represented in Fig. 4.

### Cerebellar stimulation: motor and cognitive functions

In the current literature, some studies have demonstrated the positive impact of cerebellar tDCS on motor and cognitive functions in healthy subjects with a relevant effect size of 0.71 for motor function and 0.32 for cognitive tasks [86, 87]. However, to date, the clinical translation of cerebellar tDCS in a clinical population still need to be investigated since only a few studies have been conducted. The first study, published in 2009, investigated the effect of cerebellar tDCS on cortical excitability by means of MEP [88]. The authors showed that stimulating the cerebellum could modulate M1 excitability via cerebello-cortical connectivity. Another study presented similar results with transcranial alternating stimulation more recently [89]. Other behavioral studies have shown that targeting the cerebellum could modulate motor learning, [88, 90–92], as well as cognitive functions [68]. Galea and collaborators compared the effects of cerebellar and primary motor cortex anodal stimulation on visuomotor adaptation [93]. In this clinical trial, while cerebellar stimulation promoted a faster adaptation process, M1 stimulation considerably improved retention of newly learnt visuomotor transformation [93]. Interestingly, these results demonstrate a clear distinction between the process of acquisition and retention during adaptive motor learning. In another study, the same group evaluated the effect of cerebellar stimulation on motor learning in older adults and found an improvement of adaptation with a rate similar to younger subjects [94].

### Cerebellar stimulation for stroke patients

In stroke population, Zandvliet tested the effects of a single anodal cerebellar tDCS session on standing balance in 15 patients with chronic stroke in a randomized controlled double-blind clinical trial. During stimulation, subjects performed a medio-lateral postural tracking task on a force platform. 10 patients (67%) responded to the stimulation and showed clinically relevant, even if transient, improvement of standing balance [95]. The long-term effects of repeated sessions of cerebellar tDCS still need to be determined. However, these preliminary findings are already promising, especially given the high rate of responders. Picelli and collaborators also evaluated the effects of cerebellar tDCS combined with another therapy in 20 chronic stroke patients [96]. In this double-blind RCT, tDCS was combined with transcutaneous spinal direct current stimulation on robot-assisted gait training device (5 sessions per week for two consecutive weeks). The authors tested both anodal and cathodal stimulation and evaluated the effects up to 4-week post-treatment and found a significant improvement of gait for the group who received cathodal tDCS over the contralesional cerebellar hemisphere combined with cathodal transcutaneous spinal direct current stimulation; however, this effect was not maintained at 2 nor 4-week follow-ups. Once more, this result is promising even if the effects did not last over time. Nonetheless, it is important to stress that we cannot disentangle tDCS from spinal cord stimulation’ effects as both active groups received both interventions.

Besides motor function, researchers also investigated the effects of tDCS over the cerebellum to enhance language. A recent single-case study showed that anodal right cerebellar tDCS could improve language treatment in a patient with chronic bilateral middle cerebral artery infarctions [97]. This patient received 15 sessions of tDCS coupled with spelling therapy in a double-blind, within-subject crossover design. Greater improvement with tDCS than with sham, especially for untrained words was objectified. In addition, generalization to written picture naming was only observed during tDCS. Regarding functional connectivity, clinical improvements were correlated with an increase in cerebro-cerebellar network connectivity. These results highlight the therapeutic potential of cerebellar tDCS as an adjuvant to spelling therapy in chronic stroke patients [97].

An important issue of cerebellar stimulation that may also explain some of the mixed results is the electrode montage. Recent studies on this type of stimulation targeted only a specific cerebellum hemisphere [98–100], while others positioned the active electrode over both hemispheres [92, 101]. Also, the reference electrode placement differed among studies. Possible areas of electrodes positioning include the right shoulder, buccinator muscle, supraorbital region and the vertex [102]. This wide variability of electrodes montages has an important impact on the current filed and related potential clinical effects. Indeed, these montages may induce different effects given the distinct current distribution in the cerebellum [102]. In addition, it limits the comparability of the available studies. However, computational modelling and clinical studies are still needed to investigate the impact of different montages on the effects of cerebellar stimulation [103].

In summary, cerebellar tDCS seems to be a promising tool to improve both motion and cognitive functions following stroke. However, to date, there is a critical lack of large sample RCT evaluating the long-term effects of cerebellar tDCS on both motor and cognitive functions.

## Discussion

Given the existence of different montages and electrodes positioning, finding the optimal tDCS application to improve motor performance is a challenging task. Regarding stroke, for instance, due to pathophysiological elements related with this condition, it has been suggested by different studies that anodal and cathodal tDCS are the ideal options as it increases neuroplasticity and reverts maladaptive processes, which hinder motor rehabilitation. As observed through different motor improvement scales and tests, tDCS combined with other types of techniques, such as physical therapy, and stimuli may promote even more positive results. Thus, more studies are fundamental in order to optimize these techniques and ideally validate them as alternative treatments for motor recovery.

Alternative options regarding motor recovery include Transcranial Magnetic Stimulation (TMS), pharmacotherapy, physical therapy and several others. Nonetheless, the advantages of tDCS over these methods include the ease of use, its safety, portability [42], the non-existing risk of addiction and especially, its long-term effects. Nitsche and Paulus have demonstrated that the modulating effects of both anodal and cathodal tDCS on brain tissue outlasts the duration of the stimulation [3, 6], thus prolonging the tDCS’ therapeutic effects on motor recovery and consequently facilitating the process of consolidating the neuronal network due to a possible cumulative effect after each tDCS session. On the other hand, the variation in conductivity between physical characteristics of individuals (e.g. hair, scalp and bone composition) can interfere with the current that is carried to the brain [42]. When compared with TMS, electric current induced by tDCS is not focal. Hence, it possibly stimulates not only M1, for instance, but also adjacent areas, consequently losing some precision. TMS, on the contrary, is more focal, resulting in a more specific current application. Furthermore, while TMS induces an action potential, tDCS does not induce one, but only facilitate its triggering as the constant current fields produced by tDCS are not sufficient to promote the fast depolarization required to induce an action potential in neural membranes. Therefore, tDCS is only able to decrease/increase the membranes’ threshold and thus, it modulates neural excitability.

Another strategy that has been investigated, in regard to optimizing the application of tDCS for motor recovery, is the combination between tDCS and behavioral therapies as well as the combination between tDCS and other types of stimulation, such as peripheral stimulation. Interestingly, it has been already demonstrated in different studies that combining brain stimulation with physical therapy or robotic therapies induce stronger effects than each intervention alone [104–106]. For the treatment of other clinical conditions, including Major Depressive Disorder, the combination of tDCS and behavioral therapies (cognitive trainings) have shown to provide beneficial effects [107]. The use of this combination in post-stroke patients for enhancing motor rehabilitation is still under investigation, although researchers believe that tDCS may facilitate the effects promoted by behavioral exercises [46].

Additionally, one of the combinations that have been widely explored over the years, is the use of central and peripheral stimulation concomitantly. Although there are several techniques, the combination between tDCS and TENS has been showing positive results among different studies as both interventions have been proven effective, individually, for several conditions. Therefore, the current challenge is how to optimize this combination, considering the duration of each stimulation, the interval between them, the ideal number of sessions, and other important parameters. Additionally, the real efficacy of these two types of stimulation combined is still controversial, as some studies have observed that there may be synergistic effects between them, while others do not show a relation. Hence, further research is crucial in order to investigate the mechanisms underlying the application of these types of stimulation, how effective this combination is, as well as the possible after effects.

Stimulation of the prefrontal cortex, especially the DLPFC, is also a scope of investigation for a wide variety of clinical conditions. Evidence on tDCS over the prefrontal cortex for augmenting motor performance are heterogeneous due to the diversity of stimulation parameters and protocols. While some clinical trials have suggested that the combined stimulation of M1 and DLPFC may increment motor recovery, few studies have demonstrated null effect of DLPFC stimulation on motor function outcomes. In fact, the mechanisms underlying neuron connections between the prefrontal and primary motor cortex are still insufficiently understood. Therefore, future trials investigating the functional connectivity of this brain regions are essential for a better comprehension of tDCS neurophysiological effects.

A different type of stimulation that has been explored in a few pilot studies, is the one which targets an infratentorial region, the cerebellum. Given the role of this region in fine motor movement, balance, gait, motor learning, among others, it seems to be a valuable target to stimulate in order to improve motor recovery following a stroke. Preliminary findings have shown the beneficial effects of cerebellar anodal tDCS on standing balance, while cathodal tDCS over the contralesional cerebellar hemisphere has shown to improve patients’ gait. In addition to its effects on motor functions, cerebellar tDCS may also be a valuable option to stimulate cognitive functions such as language. Research on cerebellar tDCS in stroke is still at its infancy, however, preliminary results are encouraging. However, the broad variety of electrodes montages, especially regarding the reference electrode, makes it difficult to compare exciting findings.

The stimulation techniques discussed in this review hold potential for modulating neuron networks and enhancing motor rehabilitation. In spite of the divergent results presented by clinical trials in the field, it is crucial to highlight that stimulation effects may differ among subjects, as individual characteristics alone may influence stimulation outcomes. Accordingly, while some patients may present excelling response by using traditional M1 techniques, other individuals experience greater effects on motor function through combined central and peripheral stimulation. Therefore, finding biological and neurophysiological markers of response to stimulation consists of a useful strategy for determining the most adequate intervention for each individual.

## Conclusion

In spite of the large amount of data showing tDCS as a promising adjuvant tool for motor rehabilitation, further studies are still needed. The diversity of parameters, such as current density, number of sessions, intervals between sessions and electrode montages, associated with different characteristics of the clinical populations, has generated studies with heterogeneous methodologies and controversial results. Defining the montage that enhances neuronal plasticity and reverts maladaptive process could improve patient care. The ideal montage for motor rehabilitation should be based on a patient-tailored approach that considers aspects related to the safety of the technique in that specific population and the quality of the available evidence.

## Data Availability

Not applicable.

## Abbreviations

CSF: Cerebral Spinal Fluid
DLPFC: Dorsolateral Prefrontal Cortex
EEG: Electroencephalography
LTD: Long-term Depression
LTP: Long-term Potentiation
MEP: Motor Evoked Potential
NIBS: Non-Invasive Brain Stimulation
RA: Rheumatoid Arthritis
RCT: Randomized Clinical Trials
TCI: Transcallosal Inhibition
tDCS: Transcranial Direct Current Stimulation
TENS: Transcutaneous Electrical Stimulation
TMS: Transcranial Magnetic Stimulation
